# Safety of Rapid Daratumumab Infusion: A Retrospective, Multicenter, Real-Life Analysis on 134 Patients With Multiple Myeloma

**DOI:** 10.3389/fonc.2022.851864

**Published:** 2022-03-14

**Authors:** Francesca Bonello, Serena Rocchi, Gregorio Barilà, Michela Sandrone, Marco Talarico, Elena Zamagni, Matilde Scaldaferri, Susanna Vedovato, Cecilia Bertiond, Laura Pavan, Sara Bringhen, Francesco Cattel, Renato Zambello, Michele Cavo, Roberto Mina

**Affiliations:** ^1^ SSD Clinical Trial in Oncoematologia e Mieloma Multiplo, Division of Hematology, University of Torino, Azienda Ospedaliero-Universitaria Città della Salute e della Scienza di Torino, Torino, Italy; ^2^ IRCCS Azienda Ospedaliero-Universitaria di Bologna, Istituto di Ematologia “Seràgnoli”, Bologna, Italy; ^3^ Dipartimento di Medicina Specialistica, Diagnostica e Sperimentale, Università di Bologna, Bologna, Italy; ^4^ Department of Medicine (DIMED), Hematology and Clinical Immunology Section, Padova University School of Medicine, Padova, Italy; ^5^ S.C. Farmacia Ospedaliera, A.O.U. Città della Salute e della Scienza di Torino, Turin, Italy

**Keywords:** multiple myeloma, daratumumab, rapid infusion, real life, infusion-related reactions

## Abstract

**Background:**

The anti-CD38 monoclonal antibody daratumumab is the backbone of most anti-multiple myeloma (MM) regimens. To mitigate the risk of infusion-related reactions (IRRs), intravenous daratumumab administration requires 7 hours for the first infusion and 3.5-4 hours thereafter, thus making daratumumab-containing regimens burdensome for patients and health care resources. Preliminary data suggest that a rapid (90-minute) infusion of daratumumab is safe and does not increase IRRs. The rapid schedule was adopted by our centers since 2019.

**Methods:**

We conducted an observational multi-center, real-life study to assess the safety of rapid daratumumab infusion protocol from the third administration in relapsed MM patients receiving daratumumab alone or in combination with lenalidomide-dexamethasone or bortezomib-dexamethasone. The primary endpoint was the safety of the rapid infusion protocol, particularly in terms of IRRs.

**Results:**

A total of 134 MM patients were enrolled. IRRs occurred in 7 (5%) patients and were mostly mild (6/7 of grade 1-2), with only 1 patient experiencing a grade 3 IRR. Due to the IRRs, 5 (3.7%) patients discontinued the rapid infusions and resumed daratumumab at the standard infusion rate, while 1 patient permanently discontinued daratumumab. In 4/7 patients (57%), IRRs occurred while resuming rapid daratumumab infusions after a temporary interruption (2-4 months). No other adverse event was considered related to the rapid infusion protocol.

**Conclusions:**

Our findings confirmed the safety of rapid daratumumab infusions starting from the third administration. In case of prolonged daratumumab interruption, it is advisable to resume infusions at the standard rate (3.5 hours) before switching to the rapid infusion.

## Introduction

Daratumumab is a fully human, IgG kappa monoclonal antibody targeting CD38 expressed by plasma cells. In several phase III trials, the addition of daratumumab to standard anti-multiple myeloma (MM) regimens reduced the risk of disease progression or death by 45-60% in both newly diagnosed (ND)MM and relapsed/refractory (RR)MM patients, as compared to control arms (1–6). Based on these results, the Food and Drug Administration (FDA) and European Medicines Agency (EMA) approved daratumumab as monotherapy first and then in combination with lenalidomide-dexamethasone (Dara-Rd), bortezomib-dexamethasone (Dara-Vd), pomalidomide-dexamethasone (Dara-Pd, by the FDA only), or carfilzomib-dexamethasone (Dara-Kd, by the FDA only) in the relapse setting and as first-line treatment in combination with bortezomib-melphalan-prednisone (Dara-VMP) or Rd (Dara-Rd) for transplant-ineligible patients and with bortezomib-thalidomide-dexamethasone (Dara-VTd) for transplant-eligible patients. Since its first approval for patients in later lines, the use of daratumumab has constantly increased and it currently represents the backbone of most anti-MM regimens. Virtually, all patients will receive it during their disease course, either at diagnosis or at relapse.

Similarly to other monoclonal antibodies, infusion-related reactions (IRRs) are the most frequent adverse events (AEs) experienced by patients receiving daratumumab and consist of upper respiratory tract symptoms (cough, throat irritation, nasal congestion, wheezing, or shortness of breath), chills, rash, and gastrointestinal symptoms. IRRs are the consequence of the expression of CD38 on different cells other than plasma cells, and particularly on upper airway muscle cells (7). Data from clinical trials reported an overall rate of IRRs ranging from 35% to 45%, with limited grade 3-4 events (5%) that rarely resulted in treatment discontinuation (<1%) and were mostly limited to the first infusion (≥90%) (1–6, 8).

In order to reduce the rate and severity of IRRs occurring during the first intravenous infusion of daratumumab, its infusion speed, and consequently the dose delivered, are progressively titrated for a total infusion time of approximatively 6.5 hours; then, the infusion time is reduced to 4.5 hours during the second infusion and to the standard 3.5 hours during all subsequent infusions.

In a prospective, single center study, Barr and colleagues reported for the first time that a rapid daratumumab infusion protocol (90 minutes), administered to 28 MM patients from the third infusion onwards, was safe and did not result in an increase in IRRs (9). Based on the results of this study, several medical centers have adopted the rapid infusion schedule. Here we present the results of an observational, multicenter Italian study conducted to confirm the safety and feasibility of rapid daratumumab infusion in MM patients.

## Materials and Methods

### Study Design and Patient Selection

This was a retrospective, multicenter, observational study aiming to evaluate the safety of the rapid, 90-minute daratumumab infusion in RRMM patients treated in three Italian hospitals (Azienda Ospedaliero-Universitaria Città della Salute e della Scienza di Torino, Torino; IRCCS Azienda Ospedaliero-Universitaria di Bologna, Bologna; and Azienda Ospedale-Università di Padova, Padova, Italy) after its implementation in clinical practice. A chart review of all RRMM patients receiving rapid daratumumab, either as single agent or in combination with Vd or Rd, between October 2019 and January 2021, was conducted. Patients were included in the safety analysis if they had received at least 1 rapid infusion of daratumumab. As per clinical practice, before switching to the rapid daratumumab infusion, patients had to have received the first 2 doses of daratumumab and to prove that they had tolerated the standard 200 mL/hr daratumumab infusion rate. As in the study conducted by Barr et al. in our study the rapid daratumumab infusions were administered with 20% of the total dose delivered in 30 minutes (200 mL/hr) and the remaining 80% in the following 60 minutes (450 mL/hr) (9). To limit the occurrence of IRRs, premedication was administered, as per local policy, before daratumumab infusions.

This study was conducted in accordance with the current International Conference on Harmonization Good Clinical Practice (ICH GCP) Guideline, the European Union (EU) Clinical Trials Directive, the basic principles of the Declaration of Helsinki, and local ethical and legal requirements. A protocol approved by the institutional review boards at each of the participating institutions was available before the start of this study and allowed data collection of all daratumumab-treated patients who provided written informed consent.

### Study Endpoints

The primary endpoint of this study was the safety of the rapid daratumumab infusion in terms of incidence, type, and severity of IRRs as well as of other related AEs occurring during the first 3 months from the first rapid daratumumab infusion. Daratumumab-related AEs were evaluated and graded according to the Common Terminology Criteria for Adverse Events (CTCAE, version 5.0). Secondary endpoints included the rates of IRRs according to age and comorbidities.

### Statistical Analysis

Baseline demographics, clinical characteristics, and safety data were summarized in a descriptive way and were described as either medians, ranges, or frequencies. Safety assessments were performed in all patients enrolled in this study who received at least 1 rapid daratumumab infusion. The incidences of AEs in different subgroups of patients were compared with the use of Fisher’s exact test or chi-square test, as appropriate. Based on the incidence of IRRs with the rapid infusion of daratumumab reported by previous studies, the procedure was considered safe if the incidence of daratumumab-related IRRs was lower than 5.3% (95% CI 2.7-10.2).

## Results

### Patient Characteristics

One hundred and thirty-four patients (54 from the Azienda Ospedaliero-Universitaria Città della Salute e della Scienza di Torino, 40 from the IRCCS Azienda Ospedaliero-Universitaria di Bologna, and 40 from the Azienda Ospedale-Università di Padova), who received rapid daratumumab infusions between October 2019 and January 2021, were enrolled in this study. The median age was 69 years (range 42 – 83), and 32 (24%) patients were ≥75 years old. Sixty-five (49%) patients had cardiovascular comorbidities (mainly hypertension: 40%); 8 (6%) patients suffered from chronic obstructive pulmonary disease (COPD).

Details on daratumumab regimens are summarized in **Table 1**. The majority of patients received daratumumab in combination with either Rd (102 patients, 76%) or Vd (23 patients, 17%), while 8 patients (6%) received single-agent daratumumab, and only 1 patient received daratumumab in combination with Pd.

**Table 1:** Daratumumab regimen.

Characteristics	N = 134
**Regimen**	
Single-agent daratumumab	8 (6%)
Daratumumab-Rd	102 (76%)
Daratumumab-Vd	23 (17%)
Daratumumab-Pd	1 (1%)
**Previous lines of therapy, n**	
Median (range)	1 (1 – 6)
1	101 (75.5%)
2 – 3	25 (18.5%)
>3	8 (6%)
**Premedication**	
Chlorpheniramine 10 mg, acetaminophen 1000 mg, methylprednisolone 60 mg	5 (3.5%)
Chlorpheniramine 10 mg, acetaminophen 1000 mg, dexamethasone 20 mg	89 (66.5%)
Ranitidine 300 mg, chlorpheniramine 10 mg, methylprednisolone 60 mg, acetaminophen 1000 mg, montelukast 10 mg	40 (30%)
**First rapid daratumumab infusion**	
3^rd^ dose	14 (10.5%)
4^th^ – 8^th^ dose	43 (32%)
≥9^th^ dose	77 (57.5%)

N, number; R, lenalidomide; d, dexamethasone; V, bortezomib; P, pomalidomide.

Fourteen patients (10.5%) started the rapid daratumumab infusion protocol during the first cycle after receiving the first 2 doses of daratumumab as per manufacturer’s schedule (on cycle 1, day 15), whereas the majority of patients (120, 89.5%) started the rapid daratumumab infusion protocol with a median of 4 prior cycles of daratumumab (range 0 – 33).

Fifty-eight patients (43%) had experienced a prior IRR during the first daratumumab administration, without recurrence in subsequent infusions. IRRs during the first infusion of daratumumab were all of grade 1 (41.5%) or grade 2 (58.5%) and were respiratory (48%), gastrointestinal (27.5%), or cutaneous (26%) in nature.

Before daratumumab infusion, according to local policy, all patients received premedication, which consisted of acetaminophen (1000 mg), antihistamine (ranitidine 300 mg or chlorpheniramine 10 mg), and dexamethasone (or equivalent corticosteroid) at various dosages.

### Safety

During the observation period, a total of 754 rapid daratumumab infusions were delivered to 134 patients. Overall, IRRs occurred in 7 patients (5%; **Tables 2**, **3**) during 7/754 rapid infusions, 6 of which were of grade 1 (2 patients) or grade 2 (4 patients) and were promptly resolved by administrating additional steroids and antihistamine; daratumumab was safely resumed thereafter. One patient (<1%) had a grade 3 IRR characterized by sudden hypertension and severe desaturation that required oxygen support, bronchodilators, nitrates, and high doses of hydrocortisone; this ultimately led to permanent daratumumab discontinuation. This patient had a previous history of COPD (though pulmonary function tests before starting treatment were normal); he had not experienced any IRRs during previous daratumumab infusions administered at the standard rate. Before the rapid infusion, he had received premedication with chlorpheniramine, paracetamol and dexamethasone, plus montelukast due to his history of COPD.

**Table 2:** Infusion-related reactions with rapid daratumumab infusion protocol.

Characteristics	N = 7
**Grade**	
Median (range)	2 (1 – 3)
1	2 (28.5%)
2	4 (57.25%)
3	1 (14.25%)
**Symptoms**	
Respiratory symptoms only	2 (28.5%)
Respiratory and cardiovascular symptoms	1 (14.25%)
Respiratory and cutaneous symptoms	1 (14.25%)
Other symptoms (gastrointestinal, general)	3 (43%)
**Onset**	
During the rapid daratumumab infusion	6 (85.75%)
Delayed IRR	1 (14.25%)
**Rapid daratumumab infusion at which the IRR occurred**	
1^st^	5 (71.5%)
≥2^nd^	2 (28.5%)
**Outcome**	
Rapid daratumumab infusion continued	1 (14.25%)
Discontinuation of rapid daratumumab infusion	5 (71.5%)
Permanent daratumumab discontinuation	1 (14.25%)

N, number; IRR, infusion-related reaction.

**Table 3:** Details about patients experiencing IRRs during rapid daratumumab infusion.

Patient	Age (years)	IRR at 1st standard infusion	Rapid infusion premedication	IRRs at rapid infusion	Outcome
1	64	Yes	Chlorpheniramine 10 mg, acetaminophen 1000 mg, dexamethasone 20 mg, montelukast 10 mg	Erythema, dyspnea without desaturation	Rapid infusions discontinued
2	70	No	Chlorpheniramine 10 mg, acetaminophen 1000 mg, dexamethasone 20 mg	Dyspnea with mild desaturation (sO2 91%), nasal congestion, throat irritation	Rapid infusions discontinued
3	79	No	Chlorpheniramine 10 mg, acetaminophen 1000 mg, dexamethasone 20 mg	Dyspnea with mild desaturation (sO2 89%), nasal congestion, throat irritation	Rapid infusions discontinued
4	71	No	Chlorpheniramine 10 mg, acetaminophen 1000 mg, dexamethasone 20 mg, montelukast 10 mg	Dyspnea, severe desaturation (sO2 70%), hypertension, tachycardia	Daratumumab permanently discontinued
5	68	Yes	Chlorpheniramine 10 mg, acetaminophen 1000 mg, dexamethasone 20 mg	Headache, chest oppression with normal ECG parameters	Rapid infusions discontinued
6	65	No	Chlorpheniramine 10 mg, acetaminophen 1000 mg, dexamethasone 20 mg	Delayed (2 hours after the end of the infusion) nausea, vomiting and fatigue	Rapid infusions discontinued
7	64	Yes	Ranitidine 300 mg, chlorpheniramine 10 mg, methylprednisolone 60 mg, acetaminophen 1000 mg	Nausea and vomiting during the infusion	Rapid infusions continued

IRRs, infusion-related reactions; ECG, electrocardiogram.

IRRs were characterized by respiratory (57%), cardiovascular (14%), gastrointestinal (28.5%), and general (28.5%) symptoms. In 6 out of 7 patients the IRR occurred during the infusion of daratumumab, while in 1 patient a delayed IRR occurred a few hours after the completion of the infusion.

In 5 out of 7 patients, the IRR occurred during the first rapid daratumumab infusion, when the infusion rate was increased from 200 mL/h to 450 mL/h, while in the remaining 2 patients the IRR occurred during the third and sixth 90-minute infusions, respectively. Importantly, 4 of the 7 reported IRRs (including the 2 IRRs occurred at the 3rd and 6th infusions as well as the grade 3 IRR) occurred when daratumumab administration was resumed by rapid infusion right after a treatment interruption of 2 to 4 months, either to proceed with autologous stem-cell transplantation (ASCT) or because of the limited access to the health care facilities due to the COVID-19 pandemic. Overall, 20 patients (15%) had a treatment interruption ≥2 months for the above-mentioned reasons. Among these patients, 18/20 directly resumed daratumumab with the rapid infusion schedule (IRRs in 4/18 patients, 22%), while 2/20 patients resumed daratumumab with the standard 3.5-hour schedule, before switching to the rapid schedule (no IRRs were recorded).

Among patients who experienced an IRR with the rapid daratumumab infusion protocol, no significant difference was observed between those who had previously experienced an IRR with the standard daratumumab infusion protocol and those who did not. In detail, IRRs with the rapid infusion protocol occurred in 3/58 (5.2%) patients who had previously experienced an IRR during the first standard daratumumab infusion and in 4/76 patients (5.3%) who had not (p=1.00).

Five of the 7 patients who experienced an IRR with the rapid daratumumab infusion protocol discontinued the rapid daratumumab infusion protocol and resumed the standard infusion rate (3.5 hours); 1 patient (who experienced the grade 3 IRR) permanently discontinued daratumumab, while 1 patient continued with the rapid infusions without further IRRs.

No significant difference in the incidence of IRRs was observed according to age (<75 years, 6% *vs*. ≥75 years, 3%, p=0.68). No difference in the type of administered premedication was observed in patients with *vs*. without IRRs during the rapid daratumumab infusions.

Aside from IRRs, no other daratumumab-related AEs were detected during the observation period with the rapid daratumumab infusion protocol. Of note, no patient experienced fluid overload.

## Discussion

Our multicenter study confirmed, in a large cohort of real-world patients, the safety of the rapid daratumumab infusion protocol initially reported by Barr and colleagues (9).

In our cohort, the incidence of IRRs with the rapid daratumumab infusion protocol was low (5% of patients, <1% of total IRRs during the observation period), in line with the findings of other studies, which reported an incidence of IRRs ≤5% (**Table 4**) (10–12, 14).

**Table 4:** Summary of data from main studies on rapid daratumumab infusion.

Study	Study type	Number of patients	Rapid infusion protocol	IRRs/AEs*, n (%)
Barr et al. (9)	Prospective, single center	28	90 minutes: 20% of the dose in 30 minutes (200 mL/hr), 80% in 60 minutes (450 mL/hr) after >2 infusions	- No IRRs - 1 (3%) G2 hypertension
Hamadeh et al. (10)	Retrospective, single center	53	Same as above	- 1 (2%) G2 chills and rigors
Gozzetti et al. (11)	Prospective, single center	39	Same as above	- No IRRs
Lombardi et al. (12)	Prospective, single center	25	Same as above	- 2 (8%) G1 hypotension - 2 (8%) G1 hypertension
Patel et al. (13)	Retrospective, single center	35	Same as above	- 1 (3%) G1 IRR
Gordan et al. (14)	Retrospective, single center	147	110 minutes, after >2 infusions	- No IRRs

*Refers to IRRs/AEs occurred during the rapid infusion.

IRRs, infusion-related reactions; AEs, adverse events; n, number; G, grade.

The majority of IRRs with the rapid daratumumab infusion were mild (6/7 of grade 1-2) and manageable. Permanent daratumumab discontinuation was necessary in only 1 patient due to the severity of the IRR (grade 3), while in the other patients daratumumab was resumed at the standard infusion schedule (5 patients) or maintained at the rapid schedule (1 patient).

Importantly, 4 out of 7 IRRs occurred in patients receiving the rapid daratumumab infusion after a temporary interruption (2-4 months) due to ASCT or limited hospital access during the COVID-19 pandemic. Overall, 4/18 patients (22%) resuming rapid daratumumab infusion after an interruption ≥2 months experienced an IRR.

In the GRIFFIN and CASSIOPEIA trials, respectively 3% and 11% of patients experienced an IRR when resuming daratumumab administration after ASCT (1, 15). Therefore, a possible explanation for some of the IRRs observed with the rapid daratumumab infusion protocol could be related to the re-exposure to daratumumab after a treatment break rather than to the infusion rate in itself. Hamadeh et al. recommended that patients resuming daratumumab after a treatment break ≥6 months should restart daratumumab at the standard infusion rate before switching to the rapid infusion protocol (10). The findings of our study are likely to confirm the adoption of this strategy in patients withholding daratumumab administration for more than 2 months.

Importantly, no difference in terms of IRR rate with the rapid daratumumab infusion protocol was observed in patients that had previously experienced an IRR, as compared to patients who had not. These results suggest that the rapid daratumumab infusion protocol can be adopted regardless of prior IRRs, provided that the patient is able to tolerate the standard 200 mL/hr infusion rate, which is a key criterion for starting the rapid daratumumab infusion protocol. No cardiovascular and pulmonary AEs, possibly related to fluid overload, were reported in our analysis.

A strength of our study is the inclusion of a real-life patient population referring to hematological centers, including patients with cardiovascular and pulmonary comorbidities, as well as elderly patients (24% of patients aged ≥75 years). Among the older patients, no significant increase in the rate of IRRs was observed when patients received the rapid daratumumab infusion protocol (<75 years, 6% *vs*. ≥75 years, 3%), thus confirming that older age is not an exclusion criterion for the adoption of the rapid daratumumab infusion protocol.

Compared with the standard infusion (3.5 hours), the rapid daratumumab infusion protocol (1.5 hours) is an appealing option to reduce the hospitalization time of treated patients. This is a critical point, since daratumumab administration until progression may be burdensome in terms of quality of life. In light of the COVID-19 pandemic, shorter hospital stays are particularly useful because they limit social interactions and the risk of viral transmission. The rapid infusion protocol is also advantageous for health care facilities, since it reduces the infusion time per patient by 2.3-fold, thus allowing them to increase their capacity.

A limitation of this study is the lack of a pharmacoeconomic analysis; however, previous studies conducted both in the US and Europe already reported a beneficial effect of the adoption of the rapid daratumumab infusion protocol also in financial terms (10, 12, 16).

A major advancement in the treatment of MM patients was the recent approval by the FDA and EMA of subcutaneous (SC) daratumumab, based on the results of the COLUMBA and PLEIADES trials, which confirmed similar efficacy between intravenous and SC formulations, while showing a reduction in the IRR rate with the SC formulation (17, 18). SC daratumumab has been widely implemented in the US, while, although being approved by the EMA, it is not homogeneously reimbursed in all European countries. In this view, our data could be of value in countries in which SC daratumumab is not yet approved or reimbursed.

In conclusion, our study confirmed that the rapid daratumumab infusion protocol can be implemented from the third infusion of daratumumab onwards without safety concerns, regardless of prior IRRs. Our data also suggest that, in case of a temporary interruption of the daratumumab administration (2 months or longer), daratumumab should be resumed at the standard schedule before adopting the rapid infusion protocol.

## Data Availability Statement

The raw data supporting the conclusions of this article will be made available by the authors, without undue reservation.

## Ethics Statement

A protocol approved by the institutional review boards at each of the participating institutions was available before the start of this study and allowed data collection of all daratumumab-treated patients who provided written informed consent. The participating institutions were: Azienda Ospedaliero-Universitaria Città della Salute e della Scienza di Torino (Torino, Italy), IRCCS Azienda Ospedaliero-Universitaria di Bologna (Bologna, Italy), and Azienda Ospedale-Università di Padova (Padova, Italy). The patients/participants provided their written informed consent to participate in this study.

## Author Contributions

All authors had full access to all the data in this contribution and take responsibility for the integrity of the data and the accuracy of the data analysis. Substantial contributions to the conception or design of the work: FB and RM. Acquisition, analysis, or interpretation of data for the work: All authors. Drafting of the work: FB and RM. Critical revision of the work for important intellectual content: all authors. Agreement to be accountable for all aspects of the work in ensuring that questions related to the accuracy or integrity of any part of the work are appropriately investigated and resolved: all authors. All authors contributed to the article and approved the submitted version.

## Conflict of Interest

Author SR has received honoraria from Janssen, Bristol Myers Squibb, Amgen, and GlaxoSmithKline. Author GB has served on the advisory boards for Janssen and Amgen. Author EZ has received honoraria from and served on the advisory boards for Janssen, Bristol Myers Squibb, Takeda, Sanofi, Amgen, GlaxoSmithKline, and Oncopeptides. Author SB has received honoraria from Celgene, Amgen, Oncopeptides, Janssen, and Sanofi; has served on the advisory boards for Takeda, Janssen, GlaxoSmithKline, Sanofi, Bristol Myers Squibb, and Oncopeptides; has received consultancy fees from Janssen, Takeda, Amgen, and Sanofi. Author RZ has served on the advisory boards for Celgene, Janssen, ​GlaxoSmithKline, Amgen, and Takeda. Author MC has received honoraria from Janssen, Celgene, Bristol Myers Squibb, GlaxoSmithKline, Amgen, Takeda, AbbVie, Sanofi, and Roche; has served on the advisory boards for Janssen, Celgene, Bristol Myers Squibb, GlaxoSmithKline, Amgen, Takeda, AbbVie, Sanofi, Roche, and Adaptive Biotechnologies. Author RM has received honoraria from Sanofi, Celgene, Takeda, and Janssen; has served on the advisory boards for Sanofi, Takeda, Bristol Myers Squibb, and Janssen; has received consultancy fees from Janssen.

The remaining authors declare that the research was conducted in the absence of any commercial or financial relationships that could be construed as a potential conflict of interest.

## Publisher’s Note

All claims expressed in this article are solely those of the authors and do not necessarily represent those of their affiliated organizations, or those of the publisher, the editors and the reviewers. Any product that may be evaluated in this article, or claim that may be made by its manufacturer, is not guaranteed or endorsed by the publisher.
